# Animal Models of Metabolic Disorders in the Study of Neurodegenerative Diseases: An Overview

**DOI:** 10.3389/fnins.2020.604150

**Published:** 2021-01-18

**Authors:** Andreza Fabro de Bem, Rachel Krolow, Hémelin Resende Farias, Victória Linden de Rezende, Daniel Pens Gelain, José Cláudio Fonseca Moreira, João Miguel das Neves Duarte, Jade de Oliveira

**Affiliations:** ^1^Department of Physiological Sciences, Institute of Biology, University of Brasilia, Brazilia, Brazil; ^2^Postgraduate Program in Biological Sciences: Biochemistry, Department of Biochemistry, Institute of Basic Health Sciences, Federal University of Rio Grande do Sul, Porto Alegre, Brazil; ^3^Department of Experimental Medical Science, Faculty of Medicine, Lund University, Lund, Sweden; ^4^Wallenberg Centre for Molecular Medicine, Faculty of Medicine, Lund University, Lund, Sweden

**Keywords:** Alzheimer’s disease, Parkinson’s disease, hypercholesterolemia, obesity, diabetes, rodent models, neurodegeneration

## Abstract

The incidence of metabolic disorders, as well as of neurodegenerative diseases—mainly the sporadic forms of Alzheimer’s and Parkinson’s disease—are increasing worldwide. Notably, obesity, diabetes, and hypercholesterolemia have been indicated as early risk factors for sporadic forms of Alzheimer’s and Parkinson’s disease. These conditions share a range of molecular and cellular features, including protein aggregation, oxidative stress, neuroinflammation, and blood-brain barrier dysfunction, all of which contribute to neuronal death and cognitive impairment. Rodent models of obesity, diabetes, and hypercholesterolemia exhibit all the hallmarks of these degenerative diseases, and represent an interesting approach to the study of the phenotypic features and pathogenic mechanisms of neurodegenerative disorders. We review the main pathological aspects of Alzheimer’s and Parkinson’s disease as summarized in rodent models of obesity, diabetes, and hypercholesterolemia.

## Introduction

Alzheimer’s disease (AD) and Parkinson’s disease (PD) are the two most common neurodegenerative diseases. They are chronic and progressive, and are defined by protein abnormalities, neuroinflammation (characterized by glial activation), and neuronal loss (Dugger and Dickson, 2017). Both AD and PD present genetic and sporadic forms, though the majority of cases are of sporadic type (Bekris et al., 2010; Klein and Westenberger, 2012). Vascular risk factors, such as metabolic disorders, have been linked to sporadic forms of AD and PD (Castillo et al., 2019). Several epidemiological studies have demonstrated a connection between hypercholesterolemia, obesity, and neurodegenerative disease development (Kivipelto et al., 2001, 2005; Gustafson et al., 2003; Whitmer et al., 2008; Solomon et al., 2009; Santos et al., 2017). Notably, the metabolic diseases in early life are risk factors for dementia. The epidemiological studies have shown that obesity and hypercholesterolemia in adulthood or in middle age increase the risk of dementia in the future (Kivipelto et al., 2001, 2005; Whitmer et al., 2005; Ariza et al., 2016). In the elderly, obesity and high plasma cholesterol levels are not correlated with a higher occurrence of dementia (Reitz et al., 2008; Anjum et al., 2018).

These findings were confirmed and further explored by experimental studies (Ullrich et al., 2010; de Oliveira et al., 2011; Moreira et al., 2014; Denver et al., 2018). Experimental models of obesity and hypercholesterolemia display similar brain alterations to those present in the brain of patients with neurodegenerative diseases, such as amyloid-β peptide (Aβ) accumulation, as well as abnormal tau protein and α-synuclein (Ullrich et al., 2010; Busquets et al., 2017; Han et al., 2017; Nakandakari et al., 2019). Blood-brain barrier (BBB) disruption and neuroinflammation have also been found in the brain structures of obese and hypercholesterolemic rodents (Ullrich et al., 2010; de Oliveira et al., 2014; Denver et al., 2018). Importantly, behavioral impairments related to neurodegenerative disease, particularly cognitive impairment, are evident in experimental models of obesity and hypercholesterolemia (Ullrich et al., 2010; de Oliveira et al., 2011; Moreira et al., 2014; Denver et al., 2018).

Another critical point is that the molecular mechanisms of AD and PD are still not completely known (Zeng et al., 2018), and, so far, these diseases have no cure. In this regard, experimental studies are needed to understand the structural, functional, and molecular features of these diseases and to propose therapies. The evidence is mounting that the environment has an essential role in the development of neurodegenerative diseases. It is now known that unhealthy lifestyles (including the excessive ingestion of food) combined or otherwise with specific predisposing genes are responsible for most cases of sporadic forms of AD and greatly contribute to sporadic forms of PD (Bhatti et al., 2020; Popa-Wagner et al., 2020). However, the classic models of neurodegenerative diseases, based on genetic alterations, do not encompass all the characteristics of these complex diseases (Ransohoff, 2018).

Given that animal models of metabolic disorders share most of the brain dysfunction associated with neurodegenerative disease, even in early life (Ullrich et al., 2010; Moreira et al., 2014; Paul et al., 2017a; de Oliveira et al., 2020b), they represent an interesting strategy for studying the prior events of neurodegeneration. This review contains the main findings regarding AD and PD in rodent models of metabolic diseases (in particular, obesity and hypercholesterolemia), to highlight their relevance in the study of aspects related to neurodegeneration.

## Animal Models of Alzheimer’s Disease

**FIGURE 1:**
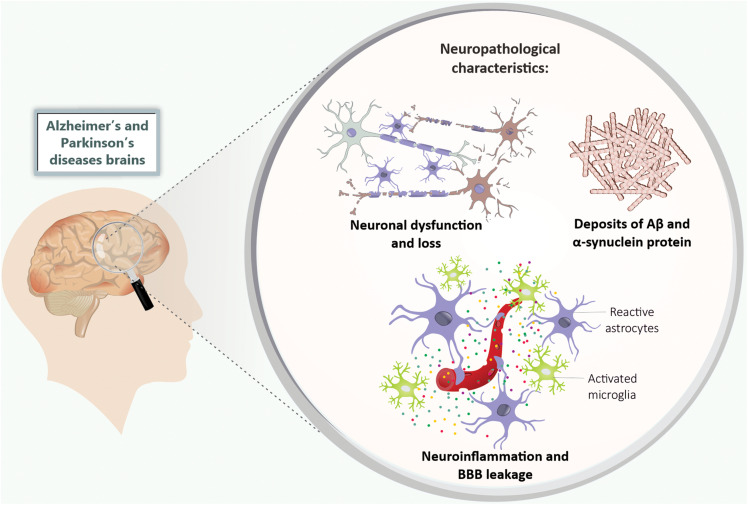
Neuropathological features of Alzheimer’s and Parkinson’s disease. The Alzheimer’s and Parkinson’s disease patients’ brains present neuronal dysfunction and loss, deposits of Aβ and α-synuclein protein, neuroinflammation (mainly characterized by microglia and astrocytes activation), and BBB leakage. Aβ, amyloid-β peptide; BBB, blood-brain barrier.

Alzheimer’s disease is clinically characterized by cognitive impairments such as memory deficits (Kelley and Petersen, 2007), as well as the irreversible decline in the number of basal forebrain cholinergic neurons and synaptic loss mainly in the hippocampus and cerebral cortex (Schliebs and Arendt, 2011; Kozlov et al., 2017). Neuropathological components of this disorder include the presence of extracellular Aβ aggregates that precipitate in amyloid plaques, and neurofibrillary tangles mainly formed by hyperphosphorylated tau protein (Hardy and Selkoe, 2002; Selkoe and Hardy, 2016; Figure 1).

Alzheimer’s disease research mainly focuses on the hypothesis of the amyloid cascade, which postulates that the characteristic neuronal damage of the disease is partly attributed to changes in Aβ metabolism (Hardy and Selkoe, 2002; Selkoe and Hardy, 2016). Aβ is the product of amyloidogenic metabolism of amyloid precursor protein (APP) by enzymes called secretases (β and γ secretases) (O’Brien and Wong, 2011). Studies indicated that the Aβ becomes toxic by forming oligomers, which ultimately result in amyloid plaques deposition, neurodegeneration, and, consequently, cognitive impairments (Ferreira and Klein, 2011; Hardy and Selkoe, 2002; Selkoe and Hardy, 2016). AD is also characterized by chronic brain inflammation and BBB disruption (Zenaro et al., 2017; Figure 1).

Mutations in genes related to APP processing result in genetic AD (early-onset) (Levy et al., 1990). However, mutations in these genes account for only a small proportion of the disease, with sporadic (late-onset) AD accounting for 99% of the cases. It is also important to highlight that the etiology of Aβ deposits in sporadic AD remains unclear in most cases (Zhang et al., 2018; Tzioras et al., 2019). Sporadic AD is considered a multifactorial and complex neurodegenerative pathology, resulting from the interaction of genetic and environmental risk factors. Several diseases have been considered risk factors for AD, among them are the metabolic diseases that are causative events for cardiovascular diseases (Campos-Peña et al., 2017). Also, the presence of apolipoprotein E (ApoE) ε4 allele (APOE4) is the most important genetic risk factor for sporadic AD (Liu et al., 2013).

Even AD being the most important cause of dementia, until now, no treatment delays the onset or progression of the disease and its pathogenesis is still not elucidated. The availability of experimental models that cover the multifaceted aspects of AD is essential to perform translational studies (Long and Holtzman, 2019; Tiwari et al., 2019). There are several experimental models of AD, including genetic-based models of amyloid pathology (mainly transgenic mice) and those involving rodents exposed to intracerebroventricular (ICV) or intrahippocampal injection of Aβ (Puzzo et al., 2015).

### Genetic-Based Mouse Models of Alzheimer’s Disease

Although none of the existing models fully reproduces the complete spectrum of AD, specific critical aspects of AD pathology and disease processes can be experimentally recreated in experimental rodent models (LaFerla and Green, 2012). Several animal models, using mice and rats mainly, have been used to create genetically altered phenocopies of human AD. Transgenic mice overproducing mutant tau and APP proteins (e.g., PDAPP and PS19 mice) and/or some of the enzymes implicated in their metabolic processing have been bred (Games et al., 1995; Hsiao et al., 1996; Oddo et al., 2003a, b; Drummond and Wisniewski, 2017).

For instance, PDAPP mice overexpress different isoforms of APP (695, 751, and 770), presenting Aβ deposits in different brain areas. The brain Aβ deposits start at 6–9 months of age and progress in an age-dependent manner (Games et al., 1995; Johnson-Wood et al., 1997; Chen et al., 2000), while behavioral alterations, such as spatial learning and memory impairments in the radial maze, appear at 3–4 months in PDAPP mice (Dodart et al., 1999; Hartman et al., 2005). This fact is a critical issue in this model, since the memory impairment does not correlate with brain Aβ deposits (Chen et al., 2000; Hartman et al., 2005). Concerning other pathogenic aspects, the PDAPP model displays an increased number of activated microglia and astrogliosis (Games et al., 1995), but the BBB integrity is intact until 16 months of age (Blockx et al., 2016).

Another example is the transgenic mouse Tg2576, which overexpresses a mutant form of APP (695) associated with the Swedish mutation (K670N, M671NL; Hsiao et al., 1996). The Tg2576 mouse better represents the connection between the formation of amyloid plaques and behavioral changes that are characteristic of AD. Around 4–5 months of age, Tg2576 mice present contextual memory deficits and an increase in the fraction of Aβ_1__–__42_ relative to Aβ_1__–__40_ (Jacobsen et al., 2006). Parenchymal Aβ plaques occur between 11 and 13 months of age (Hsiao et al., 1996). An increase in microglial density was observed in these mice when they turned 10–16-months of age (Frautschy et al., 1998). Before the formation of plaques, at 4 months of age, Tg2576 mice exhibit BBB disruption in some areas of the cerebral cortex (Ujiie et al., 2003) and in others brain areas at 8 months of age, which can be visualized by magnetic resonance imaging (MRI; Elhaik Goldman et al., 2018).

The triple-transgenic mouse model (3xTg) expresses three significant genes associated with familial AD (APPSwe, PSN1M146V, and tauP301L; Oddo et al., 2003a, b, 2005). It was designed to be an animal model for studying plaque and tangle pathology associated with synaptic dysfunction. The intracellular Aβ deposition starts at 3 months of age, while extracellular deposition of Aβ occurs in 6-month-old animals (Oddo et al., 2003b); cognitive impairments start at 4 months old (Billings et al., 2005). Other notable features of AD recreated in the 3xTg mice are neuroinflammation, synaptic dysfunction, and BBB impairment (Parachikova et al., 2010; Do et al., 2014; Belfiore et al., 2019). Although this mouse model is considered the most complete transgenic mouse model of AD available, the widespread presence of plaques and tangles are typically not observed until old age, and are not representative of human AD (Drummond and Wisniewski, 2017). In fact, the limitations to finding the AD molecular and morphology features in this transgenic animal model impair translational comparisons.

The 5xFAD mouse is an experimental model designed to reduce the time before amyloid plaques are formed. This transgenic mouse combines five mutations: Swedish mutation (APP KM670/671NL), London (V717l), Florida (APP I716V), L286V in PSN1, and M146L. The brain Aβ_1__–__42_ levels increase as early as 1.5 months of age and an AD-like amyloid pathology occurs at 2 months of age, while other available mouse models display amyloid deposition in the brain parenchyma between 3 and 12 months after birth (Oakley et al., 2006). In addition, these mice present spatial memory and learning deficits at 4–6 months of age (Oakley et al., 2006; Ohno, 2009).

PS19 mice expressing the P301S mutant form of human microtubule-associated protein Tau (MAPT) is also an AD mouse model (Yoshiyama et al., 2007). PS19 mice develop filamentous tau lesions at 6 months of age. Tangle pathology is accompanied by microgliosis and astrocytosis, but not by amyloid plaques (Yoshiyama et al., 2007). Interestingly, hippocampal synaptic dysfunction and loss were detected before fibrillary tau tangles emerged in the brains of these mice (Yoshiyama, 2008).

It is important to mention that metabolic parameters have been investigated in the AD animal models. For example, a glucose homeostasis impairment was demonstrated in the 3xTg-AD mice, which occurred in an age-dependent manner (Vandal et al., 2015).

One critical point in using these transgenic mouse models of AD is that they recapitulate the early-onset (familial) form of AD, which accounts for only 1% of cases. Therefore, these models may still present an incomplete perspective of the pathology (Kitazawa et al., 2012; Zou et al., 2014).

On the other hand, E4FAD and E3FAD mouse models, which are crosses between the 5xFAD mice and mice expressing APOE4 and APOE3 human isoforms, represent an effort to replicate sporadic AD. However, the E4FAD and E3FAD mice display less severe phenotypes compared with the 5xFAD mice. The brain Aβ accumulation appears from 2 to 6 months of age and is more intense in the brains of E4FAD mice (Youmans et al., 2012). Both E4FAD and E3FAD mice showed reactive microglia and dystrophic astrocytes at 6 months of age, but in E4FAD mice, the microglial reactivity was higher than in E3FAD mice (Rodriguez et al., 2014). Moreover, E4FAD mice exhibited more severe age-dependent memory deficits than E3FAD mice (Liu et al., 2015). Table 1 summarizes the main findings regarding AD-like pathology in the main transgenic AD mice models.

**TABLE 1:** Summary of the main alterations observed in the transgenic mouse models of Alzheimer’s disease.

Animal model	Gene mutation	Main Alteration	Age (months)	References
PDAPP mouse	APP (695, 751, and 770)	– ↓Spatial learning – Memory impairments	3–4	Dodart et al., 1999; Hartman et al., 2005
		– ↑ Aβ deposition	6–9	Games et al., 1995; Johnson-Wood et al., 1997; Chen et al., 2000
		– Increased activation of microglia and astrogliosis		Games et al., 1995
		– Intact BBB integrity	16	Blockx et al., 2016
Tg2576 mouse	APP (695) + Swedish mutation (K670/M671NL)	– Contextual memory deficits – ↑ Fraction of Aβ_1__–__42_ to Aβ_1__–__40_	4–5	Jacobsen et al., 2006
		– ↑ Parenchymal Aβ plaques	11–13	Hsiao et al., 1996
		– ↑ Microglial density	10–16	Frautschy et al., 1998
		– ↑ BBB disruption in the cerebral cortex and other brain areas	4 8	Ujiie et al., 2003 Elhaik Goldman et al., 2018
3xTg mouse	APP Swedish (K670/M671NL) + PSEN1 (M146V) + MAPT P301L	– ↑ Intracellular Aβ deposition	3	Oddo et al., 2003b
		– Cognitive impairments	4	Billings et al., 2005
		– ↑ Extracellular Aβ deposition – ↑ Amyloid plaques – ↑ BBB permeability	6	Oddo et al., 2003b
		– ↑ Neuroinflammation		Parachikova et al., 2010; Belfiore et al., 2019
5xFAD mouse	APP Swedish (KM670/671NL) + London (V717l) + Florida (APP I716V) + PSN1 (L286V) + M146L	– ↑ Levels of Aβ_1__–__42_ in the brain	1.5	Oakley et al., 2006
		– AD-like amyloid pathology	2	Oakley et al., 2006
		– Spatial memory and learning deficits	4–6	Oakley et al., 2006; Ohno, 2009
PS19 mouse	MAPT P301S	– Filamentous tau lesions	6	Yoshiyama et al., 2007, Yoshiyama, 2008
		– Hippocampal synaptic dysfunction and loss – Neuroinflammation	3	
E3FAD mouse	APP Swedish + London + Florida + PSN1 (L286V) + M146L + APOE3	– Aβ accumulation – Neuroinflammation	2–6 6	Youmans et al., 2012 Rodriguez et al., 2014
E4FAD mouse	APP Swedish + London + Florida + PSN1 (L286V) + M146L + APOE4	– Aβ accumulation – Neuroinflammation	2–6 6	Youmans et al., 2012 Rodriguez et al., 2014

*AD, Alzheimer’s disease; APP, amyloid precursor protein; Aβ, amyloid-β peptide; PSN1, presenilin 1; BBB, blood-brain barrier. (↑) Increased, (↓) decreased.*

### Intracerebroventricular Injection of Aβ in Rodents

It takes some time to recreate the features of AD using transgenic mice models, because the increase in Aβ levels, amyloid plaque formation, and behavioral impairments appear typically from 6 months of age (Games et al., 1995; Hsiao et al., 1996; Sturchler-Pierrat et al., 1997; Dewachter et al., 2000). Therefore, another interesting experimental tool with which to study Aβ toxicity is ICV and intrahippocampal injections of Aβ peptides (Flood et al., 1991; Piermartiri et al., 2010; Ferreira and Klein, 2011). Here, studies have demonstrated spatial learning and memory deficits induced by ICV administration of aggregated Aβ_1__–__40_ or Aβ_1__–__42_ (Yamada et al., 1999; Yan et al., 2001; Jhoo et al., 2004; Yamaguchi et al., 2006; Prediger et al., 2007) in rodents after only a few days/weeks of administration. Cognitive impairments in rodents exposed to aggregated Aβ_1__–__40_ were associated with synaptic loss and cell death in the hippocampus and prefrontal cortex (Prediger et al., 2007; Piermartiri et al., 2010; Figueiredo et al., 2011; Santos et al., 2012). Furthermore, ICV injection of aggregated Aβ_1__–__40_ or Aβ_1__–__42_ led to an increase in the hippocampal concentration of the proinflammatory cytokine, interleukin (IL)-1β (Yan et al., 2001; Minogue et al., 2003), as well as microglial activation (Clarke et al., 2007; Medeiros et al., 2007; Figueiredo et al., 2011).

Another approach is the ICV administration of soluble Aβ oligomers (AβOs). These are potent neurotoxins derived from Aβ_1__–__42_, which can be found in AD brains (Lambert et al., 1998; Ferreira and Klein, 2011). The ICV infusion of AβOs has been shown to cause synaptic loss in the hippocampus and memory impairment related to AD in mice (Ledo et al., 2013; Figueiredo et al., 2013). Moreover, mice injected by the ICV route with AβOs presented hippocampal activation of microglia and astrocytes, as well as brain increased tumor necrosis factor α (TNF-α), IL1β, and IL-6 levels (Ledo et al., 2013; Brkic et al., 2015).

In both models, that is, mice ICV injected with aggregated Aβ or AβOs, BBB integrity was not entirely explored. With regard to the ICV injection of AβOs, a loss of blood – CSF barrier integrity in the choroid plexus was observed in mice (Brkic et al., 2015). On the other hand, C57BL/6 mice injected with an aggregated form of Aβ_1__–__40_ did not present changes in the hippocampal immunoreactivity of aquaporin-4 (AQP-4), a putative marker of edema and BBB leakage (de Oliveira et al., 2014). More studies are needed to better describe this aspect in these AD experimental models. Another critical point is that it is difficult to observe amyloid plaques in the brain in this particular model (Kim et al., 2016). The heterogeneity of the peptide samples is also a problem with the application of Aβ in rodent models (Kasza et al., 2017).

## Animal Models of Parkinson’s Disease

Parkinson’s disease is the second most prevalent neurodegenerative disease after AD, and the most common movement disorder (Obeso et al., 2017). The main features of PD are dopaminergic neuronal loss in the substantia nigra pars compacta (SNpc) and dopamine depletion in the striatum (Poewe et al., 2017), with the presence of intracytoplasmic inclusions called Lewy bodies, which are composed mainly of misfolded α-synuclein (Spillantini et al., 1998). Neuroinflammation and BBB disruption have also been considered to be pathogenic features of PD (Collins et al., 2012; Wang et al., 2015; Guzman-Martinez et al., 2019; Figure 1).

Clinically, PD is characterized by motor symptoms such as resting tremor, bradykinesia, rigidity, and loss of postural reflex. These result from dopaminergic degeneration of the nigrostriatal pathway (Magrinelli et al., 2016). This neuropathology is also associated with non-motor symptoms, for example anxiety, depression, and cognitive impairments (dementia; Franke and Storch, 2017).

Parkinson’s disease can be genetic or sporadic (Singleton et al., 2013). It is known that the development of the disease occurs due to genetic susceptibility associated with environmental risk factors (Chen and Ritz, 2018). Exposure to heavy metals, fungicides, and pesticides (e.g., rotenone and paraquat) have been associated with the development of the disease (Ball et al., 2019). In addition, in the past few years, it has been suggested that metabolic disorders may be causative events of PD (Limphaibool et al., 2018; Nam et al., 2018; Alecu and Bennett, 2019).

Because PD occurs mainly as a sporadic form, experimental models based on compounds’ neurotoxicity are useful for the study of this neuropathology. For instance, the 6-hydroxydopamine (6-OHDA) animal model of PD has been used in the PD research field since 1968 (Ungerstedt, 1968). The 6-OHDA is an analog of dopamine and norepinephrine and is endogenously produced through the hydroxylation of dopamine (Tieu, 2011). This neurotoxin is unable to cross the BBB; therefore, the only way to expose the brain to neurotoxic actions of this substance is through stereotaxic surgery (Duty and Jenner, 2011). Bilateral injection of 6-OHDA into the substantia nigra (SN) of rats has caused anterograde degeneration of the nigrostriatal dopaminergic system, leading to akinesia and high mortality. The first experimental model of PD was generated thus (Ungerstedt, 1968). Specifically, after stereotaxic injection, 6-OHDA is removed from the extracellular space by dopamine or noradrenaline membrane transporters and stored in catecholaminergic neurons. Inside these neurons, 6-OHDA undergoes both enzymatic degradation by monoamine oxidase A (MAO-A) and auto-oxidation, generating several cytotoxic species that lead to neuronal damage (Soto-Otero et al., 2000). Many studies have demonstrated that brain 6-OHDA injections are associated with decreased locomotor activity, reduced tyrosine-hydroxylase (TH)-positive neurons, and brain oxidative stress in mice (Ungerstedt, 1968; Simola et al., 2007; Ozsoy et al., 2015). The 6-OHDA experimental models of PD are also associated with neuroinflammation, that is, microgliosis and astrogliosis (Walsh et al., 2011; Gasparotto et al., 2017), as well as BBB disruption (Carvey et al., 2005). Unilateral injections of 6-OHDA into the striatum or the medial forebrain bundle induced an increased BBB permeability to FITC-labeled albumin in the SN and striatum (Carvey et al., 2005). One limitation of 6-OHDA-based models is that they do not cause changes in α-synuclein expression or deposition, and for this reason they are more correctly referred to as models of “Parkinson-like” dopaminergic denervation or, simply, dopaminergic denervation.

Other rodent models of PD have been developed through exposure to 1-methyl-4-phenyl-1,2,3,6-tetrahydropyridine (MPTP). It was demonstrated that MPTP itself is not toxic; however, as a lipophilic compound, it passes through the BBB. Once in the brain, the molecule is rapidly converted into its toxic metabolite, 1-methyl-4-phenylpyridinium or MPP+, by monoamine oxidase B (MAO-B; Langston, 2017). Studies performed with rodents showed a reduction of locomotion and rearing in the open field task for several weeks after the subcutaneous and intraperitoneal administration of MPTP (Fredriksson et al., 1999; Fornai et al., 2005). In contrast, other studies reported no change in locomotion and even hyperactivity in mice after peripheral exposure to MPTP (Tomac et al., 1995; Chia et al., 1996; Luchtman et al., 2009). In a chronic model of intraperitoneal MPTP exposure, mouse dopaminergic neurons presented α-synuclein-positive inclusions and secondary lysosomes filled with proteinaceous debris and lipid droplets, which resemble deposits in the brains of PD patients (Wu et al., 2002). Yazdani et al. (2006) showed that the infusion of MPP+ into the left cerebral ventricle of rats destroyed dopaminergic neurons in the nigrostriatal pathway. Another study indicated that the presence of MPP+ is related to inflammatory reaction along with the infiltration of T-cells into the SN and striatum and activation of the microglia and increased gene expression of proinflammatory cytokines such as IL-1β, interferon γ (INFγ), and TNFα in those brain regions (Kurkowska-Jastrzebska et al., 2009). The inflammation induced by MPTP treatment seems to cause BBB failure. Mice exposed to MPTP intraperitoneal injection presented less TH-positive dopaminergic neurons, which was related to an increase in leakage of Evan’s blue dye and FITC-albumin into the striatum. The striatum BBB disruption in the MPTP mouse model was also characterized by a reduction in the tight junctions’ proteins content (Chen et al., 2008).

Rotenone (pesticide), paraquat (herbicide), and maneb (fungicide) exposure have been considered a possible environmental cause of PD (Hatcher et al., 2008). Epidemiological studies have shown that exposure to agrochemicals increases the risk of PD (Narayan et al., 2017; Pouchieu et al., 2018). The administration of agrochemicals in rodents has been used to study the mechanisms underlying PD pathogenesis. These compounds can cross the BBB and affect the dopaminergic system (Bastías-Candia et al., 2019). For example, rotenone and paraquat are known to cause dopaminergic degeneration in mice (McCormack et al., 2002; Drechsel and Patel, 2008). One advantage of rotenone administration as a PD model is that the agrochemical mimics the chronic progression of PD patients, while other metabolite exposure results only in acute damage of dopaminergic neurons (von Wrangel et al., 2015). Cannon et al. (2009) demonstrated that rotenone-treated animals presented bradykinesia, postural instability, and/or rigidity. The authors also observed the presence of α-synuclein positive aggregates in the dopamine neurons of SN. Rotenone exposure is also associated with neuroinflammation. Martinez et al. (2017) reported that the chronic administration of intragastric rotenone in mice caused progressive nigral degeneration and neuroinflammation.

Another tool used to study PD is the intracerebral administration of α-synuclein pre-formed fibrils (PFFs) in mice. Usually, the administration of PFFs is performed with unilateral and intrastriatal injection. Specifically, this model has been used to study the mechanisms by which α-synuclein aggregates spread throughout the brain. In addition to the broad spread of pathological α-synuclein deposition, neuronal loss, neuroinflammation, and some behavioral deficits were also observed in the mice injected with PFFs (Luk et al., 2012; Gordon et al., 2018; Chung et al., 2019; Earls et al., 2019).

It is also important to mention that genetic mouse models are used to study PD; however, they are heterogeneous, and no perfect model exists (Fleming et al., 2005; Bogaerts et al., 2008). One example is A53T mice that overexpress human α-synuclein with a PD-associated mutation (A53T; Giasson et al., 2002). In an A53T mouse, the human-specific soluble α-synuclein expression increases in the brain between 2 and 6 months of age and remains constant after 12 months, resulting in the dispersal of α-synuclein aggregates throughout the cortex, hippocampus, brain stem, and cerebellum. The onset of motor symptoms is variable and generally appears at 9–10 months of age (Lee et al., 2002; Paumier et al., 2013).

Although there are many animal models of PD, none of them accurately represent all the characteristic events of the pathogenesis of this disease (Dawson et al., 2010). Table 2 summarizes the characteristics of the main PD rodent models.

**TABLE 2:** A summary of the main findings in toxin-induced rodent models of Parkinson’s disease.

Toxin	Administration methods	Main alteration	References
6-OHDA	Bilateral injection into the SN	– Anterograde degeneration of the nigrostriatal dopaminergic system – Akinesia	Ungerstedt, 1968
	Unilateral injections into the right medial striatum forebrain bundle	– ↓ Locomotor activity – ↓TH-positive dopaminergic neurons – Oxidative stress	Ozsoy et al., 2015
		– BBB disruption	Carvey et al., 2005
		– Neuroinflammation (microgliosis and astrogliosis)	Walsh et al., 2011; Gasparotto et al., 2017
MPTP	Subcutaneous and intraperitoneal injections	– ↓ Motor activity and locomotion	Fredriksson et al., 1999; Fornai et al., 2005
		– = Locomotion	Tomac et al., 1995; Chia et al., 1996; Luchtman et al., 2009
		– ↑ Locomotion	Tomac et al., 1995; Chia et al., 1996; Luchtman et al., 2009
		– ↑α-synuclein-positive inclusions in neurons – Lysosomes filled with proteinaceous debris and lipid droplets	Wu et al., 2002
		– BBB failure (↓tight junctions’ proteins) – ↓ TH-positive dopaminergic neurons – ↑ Gliosis	Chen et al., 2008
	Infusion of MPP+ into the left cerebral ventricle	– ↓ Dopaminergic neurons	Yazdani et al., 2006
		– ↑ Infiltration of T cells – ↑ Activation of the microglia – ↑ Gene expression of IL-1β, INFγ, TNF-α	Kurkowska-Jastrzebska et al., 2009
Agrochemicals (rotenone, paraquat, and maneb)	Intraperitoneal injections	– ↓ TH immunoreactivity in neurons – Dopaminergic degeneration	McCormack et al., 2002; Drechsel and Patel, 2008; von Wrangel et al., 2015
		– Bradykinesia – Postural instability – Rigidity – ↑α-synuclein positive aggregates – Neuroinflammation	Cannon et al., 2009
	Intragastric administration	– ↑ Nigral degeneration – Neuroinflammation	Martinez et al., 2017
α-synuclein PFF	Intracerebral administration	– Pathological α-synuclein deposition – Neuronal loss – Neuroinflammation – Behavioral deficits	Luk et al., 2012; Gordon et al., 2018; Chung et al., 2019; Earls et al., 2019

## Metabolic Diseases as a Risk Factor for Neurodegenerative Diseases

Metabolic disorders, e.g., obesity, diabetes, hypercholesterolemia, and hypertension, are the main risk factors for cardiovascular disease. The link between metabolic disorders and the future risk of dementia has been reported in several studies (Beydoun et al., 2008; Fitzpatrick et al., 2009; Hassing et al., 2009). In the past few decades, hypercholesterolemia, that is, high levels of blood cholesterol and obesity have been connected to neurodegenerative diseases development (Kivipelto et al., 2001, 2005; Gustafson et al., 2003; Whitmer et al., 2008; Solomon et al., 2009; Santos et al., 2017). It is postulated that one of the mechanisms behind this connection is high levels of blood cholesterol or free fatty acids (FFA) inducing deregulation of lipid metabolism and altering the permeability of the BBB, leading to neuroinflammation and cognitive decline (Takechi et al., 2013; Paul and Borah, 2017; Paul et al., 2017b).

In their pioneering evidence, Sparks et al. (1990) pointed out that the brains of non-demented individuals with coronary artery disease, a condition strictly related to hypercholesterolemia, presented amyloid plaques. The same research group observed that hypercholesterolemia induced increased intracellular Aβ deposition in the hippocampus and cerebral cortex of rabbits fed a high cholesterol diet for 4, 6, and 8 weeks (Sparks et al., 1994). Furthermore, Refolo et al. (2000) showed a positive correlation between the plasma cholesterol levels and the content of the Aβ brain when treating a transgenic mouse model of AD with a hypercholesterolemic diet. Hypercholesterolemic rabbits and individuals with cardiovascular diseases also displayed neuroinflammation, and BBB increased permeability (Streit and Sparks, 1997; Sparks et al., 2000).

It has been demonstrated that obesity contributes to impaired cognitive performance and dementia (Beydoun et al., 2008; Nguyen et al., 2014; Pegueroles et al., 2018; Hou et al., 2019). An earlier 18-year follow-up conducted by Gustafson et al. (2003) suggested that overweight in old age is a risk factor for dementia, particularly AD. Moreover, Whitmer et al. (2008) reported that central obesity in midlife increases the risk of dementia regardless of diabetes and cardiovascular comorbidities. It is worth noting that the vascular and inflammatory effects of obesity may play a role in the development of neurodegenerative diseases such as AD (Naderali et al., 2009; Businaro et al., 2012; Walker and Harrison, 2015). Relatedly, obese women presented a decrease in BBB function (Gustafson et al., 2007) while bariatric surgery reversed obesity and reduced hypothalamic gliosis in women (van de Sande-Lee et al., 2020).

Insulin is mostly known for its role in clearing glucose from the circulation. The absence of insulin production and release in type 1 diabetes (T1D) and the poor action of insulin on target cells (insulin resistance) in type 2 diabetes (T2D) result in hyperglycemia. Poorly controlled glycemia in diabetes impacts the cerebrovascular system and BBB integrity and is a risk factor for AD (Vagelatos and Eslick, 2013). However, the brains of AD patients showed downregulated insulin receptors, pointing toward a role of neuronal insulin resistance in AD etiology (Steculorum et al., 2014; de la Monte, 2017), and so several clinical trials have focused on the administration of insulin to treat or prevent dementia (Lee et al., 2018).

Metabolic disorders are also related to PD. Some findings have suggested a prospective association between plasma cholesterol or a history of hypercholesterolemia and PD risk (de Lau et al., 2006; Simon et al., 2007; Hu et al., 2008; Hu, 2010). Some evidence has also pointed to obesity, diabetes, and cerebrovascular risk factors as contributors to PD development (Chen et al., 2014; De Pablo-Fernandez et al., 2018; Kummer et al., 2019), though the correlation is not yet well established; epidemiological and clinical studies present controversial data. For instance, one study found an inverse association between plasma cholesterol levels and PD clinical progression (Huang et al., 2011).

Taking into account the fact that (i) metabolic conditions such as obesity, diabetes, and hypercholesterolemia are factors that increase the individual risk of developing AD and PD; and (ii) rodent models of obesity, diabetes, and hypercholesterolemia present all the main hallmarks of AD and PD, we propose these experimental animal models as strategic tools to study neurodegeneration. The overlap of pathological features between metabolic and neurodegenerative disorders supports a mechanistic connection among these conditions that needs to be better understood.

### Animal Models of Hypercholesterolemia and Brain Dysfunction

#### Diet-Induced Hypercholesterolemia in Rodents and Brain Effects

Hypercholesterolemia can occur by genetic origin or due to a high intake of cholesterol (Cha and Park, 2019). There are many different types of experimental models of diet-induced hypercholesterolemia. Studies have used different diets and periods of exposure (Ullrich et al., 2010; Moreira et al., 2014; Paul et al., 2017a).

Diet-induced hypercholesterolemia appears to be associated with memory damage. Swiss mice fed a high cholesterol diet (1.25% cholesterol and 20% fat) for 2 months displayed short-term memory impairment (Moreira et al., 2014). Also, exposure to a high cholesterol diet has led to severe spatial learning and long-term memory deficits in rats (Ullrich et al., 2010).

The cholinergic system is critically important for memory, learning, attention, and other higher brain functions. Evidence has indicated that hypercholesterolemia has an impact on cholinergic functions in the CNS. Ullrich et al. (2010) showed that memory decline was associated with loss of choline acetyltransferase (ChAT)-positive neurons (i.e., cholinergic neurons) in the basal nucleus of Meynert, reduction of acetylcholine levels in the cerebral cortex, and an increase in cortical Aβ_1__–__42_ levels in hypercholesterolemic rats. In line with this, we previous reported increased acetylcholinesterase (AChE) activity in the hippocampus and prefrontal cortex of mice fed a high cholesterol diet (Moreira et al., 2014).

Mice exposed to a high cholesterol diet also presented motor alterations characteristic of PD. High levels of plasma cholesterol in mice caused akinesia, catalepsy, and reduced swimming performance, and were associated with a decrease in TH-positive neurons and dopamine levels in the striatum (Paul et al., 2017a). Interestingly, hypercholesterolemia increased the neurotoxicity induced by MPTP in mice. Hypercholesterolemic mice treated with MPTP exhibited a more severe loss of dopaminergic neurons in the SN and reduced striatal levels of dopamine than those treated with MPTP only (Paul et al., 2017b).

Experimental evidence supports the notion that BBB disruption and further neuroinflammation underlying hypercholesterolemia trigger brain dysfunction (Ullrich et al., 2010; de Oliveira et al., 2014; Paul and Borah, 2017; Chen et al., 2018). Ullrich et al. (2010) observed BBB disturbance in the hypercholesterolemic rats’ cortex that was visualized by increased leakage of IgG and microgliosis. Mice fed a high cholesterol diet also displayed BBB disruption in brain regions (e.g., the hippocampus, cerebral cortex, SN, and striatum) associated with neuroinflammation (astrogliosis and microgliosis; Paul and Borah, 2017; Paul et al., 2017b). The brain inflammation in rodents fed a high cholesterol diet was also related to increased levels of cytokines such as TNF-α, IL1-α, IL-1β, and IL-6 (Ullrich et al., 2010; Chen et al., 2018).

Additionally, a previous experimental study indicated that exposure to a high cholesterol diet worsened the outcomes and accelerated the disease course in an animal model of AD. Specifically, Refolo et al. (2000) observed that exposure to a high cholesterol diet increased Aβ accumulation and accelerated the AD-related pathology in a double-mutant PSAPP. The authors demonstrated a positive correlation between the levels of plasma cholesterol and cerebral Aβ content.

Notably, rodents exposed to a high cholesterol diet present innumerable pathogenic characteristics of neurodegenerative diseases. Notably, the brain dysfunction in these rodent models of hypercholesterolemia occurred early in life (Table 3). Therefore, we propose that rodents fed a high cholesterol diet are a useful model with which study the mechanisms that lead to neurodegeneration.

**TABLE 3:** A summary of evidence linking high cholesterol diets exposure in rodents to cerebral alterations associated with neurodegenerative diseases.

Diet composition (cholesterol)	Diet duration	Animal (age-months)	Main alteration	References
1.25%+ 20% fat	8 weeks	Mice (3)	– Memory deficits – ↑ AChE activity in PFC and hippocampus	Moreira et al., 2014
5%	5 months	Rats (6)	– Memory deficits – ↓ ChAT positive neurons in cerebral cortex – ↓ Acetylcholine levels in cerebral cortex – ↑ Aβ_1__–__42_ immunocontent – BBB disruption in cerebral cortex – Neuroinflammation	Ullrich et al., 2010
	12 weeks	Mice (2)	– Memory deficits – Disruption of BBB – Neuroinflammation	Paul and Borah, 2017
			– Motor alterations (akinesia, catalepsy, and ↓ swimming performance) – ↓ TH-positive neurons in striatum – ↓ Dopamine levels in striatum – Neuroinflammation	Paul et al., 2017a
	14 months	Mice (2)	– ↓ Dopaminergic neurons in substantia nigra – ↓ Dopamine levels in striatum – Neuroinflammation	Paul et al., 2017b
3%	8 weeks	Mice (6)	– Disruption of BBB – Neuroinflammation	Chen et al., 2018

*AChE, acetylcholinesterase; Aβ, amyloid-β peptide; BBB, blood-brain barrier; ChAT, choline acetyltransferase; PFC, prefrontal cortex; TH, tyrosine-hydroxylase. (↑) Increased, (↓) decreased.*

#### LDLr^–/–^ Mice as a Model for the Study of Neurodegeneration and Memory Impairments

The main form of genetic hypercholesterolemia is familial hypercholesterolemia, which is very common in the general population (Santos et al., 2016; Fairoozy et al., 2017). This metabolic disorder is caused by mutations in the low-density lipoprotein receptor (LDLr) gene, an important molecule in cholesterol metabolism (Brown and Goldstein, 1986, 2006; Soutar and Naoumova, 2007). Familial hypercholesterolemia is closely related to the development of atherosclerotic cardiovascular disease (Austin et al., 2004). Individuals suffering from familial hypercholesterolemia also display a high incidence of cognitive impairments (Zambon et al., 2010; Ariza et al., 2016).

Similarly, LDLr knockout (LDLr^–/–^) mice, a mouse model of human familial hypercholesterolemia, display learning and memory deficits (Mulder et al., 2004; de Oliveira et al., 2011). Mulder et al. (2004) reported that 11-month-old LDLr^–/–^ display spatial memory impairment in the Morris water maze task and working memory damage in T-maze spontaneous alternation analysis. We have observed that LDLr^–/–^ present spatial and working memory decline as early as 3 months of age (de Oliveira et al., 2011, 2020a).

Memory deficits in LDLr^–/–^ mice have been linked to synaptic and neuronal dysfunction. Synaptic density reduction in the hippocampus of 11-month-old LDLr^–/–^ mice has been demonstrated (Mulder et al., 2007). We recently showed a high immunoreactivity of caspase-3 protein in the hippocampal and prefrontal cortex neurons of 3-month-old LDLr^–/–^ mice (de Oliveira et al., 2020a). Neuronal damage and synaptic dysfunction have also been associated with impaired hippocampal neurogenesis in LDLr^–/–^ mice (Mulder et al., 2007; Engel et al., 2019). However, these neuronal changes were not linked with the overproduction of Aβ, since LDLr^–/–^ mice did not exhibit alterations in Aβ_1__–__42_ content in the hippocampus and prefrontal cortex (de Oliveira et al., 2020a). On the other hand, LDLr^–/–^ mice were more susceptible to Aβ ICV neurotoxicity (de Oliveira et al., 2014).

We have revealed increased activity of AChE and antioxidant disturbance in the brain areas of 3-month-old LDLr^–/–^ mice (Moreira et al., 2012; de Oliveira et al., 2014), while treatment with donepezil, an anticholinergic drug, reversed the memory decline in these hypercholesterolemic mice (Lopes et al., 2015). Importantly, neuroinflammation characterized by astrogliosis was visualized in the hippocampus of 3-month-old LDLr^–/–^ mice. The increased number of astrocytes in the hippocampus of LDLr^–/–^ mice was associated with the increased immunoreactivity of AQP-4, which indicates BBB dysfunction (de Oliveira et al., 2014). Specifically, the increased expression and content of AQP-4, a bidirectional water channel found in astroglial foot processes, and endothelial cells can indicate edema and neurotoxicity (Thomas-Camardiel et al., 2005).

**FIGURE 2:**
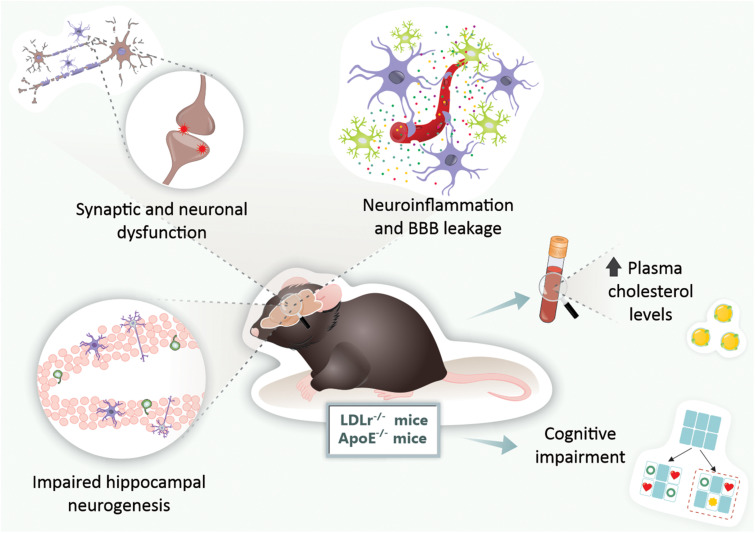
Brain alterations found in genetic mouse models of hypercholesterolemia. In LDLr^–/–^ and ApoE^–/–^ mice, both genetic mouse models of hypercholesterolemia, were observed increased levels of plasma cholesterol, BBB disruption, neuroinflammation, synaptic and neuronal dysfunction, impaired neurogenesis, and ultimately, cognitive impairments. ApoE^–/–^, apolipoprotein E knockout mice; BBB, blood-brain barrier; LDLr^–/–^, low-density lipoprotein receptor knockout mice.

We also submitted 3-month-old wild-type and LDLr^–/–^ mice to a high cholesterol diet for 30 days, and we observed that the BBB leakage was even more intense. The hippocampus and prefrontal cortex BBB dysfunction in LDLr^–/–^ mice was associated with cognitive decline, while C57BL/6 wild-types fed a high cholesterol diet exhibited impairments in the BBB but not in cognition. In addition, LDLr^–/–^ mice displayed intense astrogliosis, increased microvessel content, and decreased levels of IL-6 in the hippocampus (de Oliveira et al., 2020b). Figure 2 presents the main brain alterations found in the LDLr^–/–^ mice.

#### ApoE^–/–^ Mice as a Model for the Study of Neurodegenerative Diseases

Apolipoprotein E was first discovered by Shore and Shore (1974) in very-low-density lipoprotein (VLDL). ApoE in the periphery is principally produced by the hepatocytes but is also expressed by other cells (Kurano et al., 2011). In the brain, ApoE is synthesized mainly by astrocytes, and it plays a vital role in neuronal repair and maintenance (Huang et al., 2004). In the CNS, ApoE serves as the primary carrier protein of lipids, redistributing and mobilizing cholesterol between cells. These ApoE functions in cholesterol transport are essential for maintaining myelin and neuronal membranes (Leduc et al., 2010).

Apolipoprotein E knockout (ApoE^–/–^) mice were first designed for atherosclerosis pathogenesis studies, because they exhibit a 5- to 10-fold increase in plasma cholesterol levels (Zhang et al., 1992). Currently, ApoE^–/–^ mice have also been used for studying the pathophysiology of neurological diseases, including neurodegenerative ones (Yin and Wang, 2018).

Previous studies have demonstrated that ApoE^–/–^ mice display a disruption in spatial learning as early as 3 months and working memory impairment at 6–8 months. Both were Morris water maze protocols (Gordon et al., 1995; Masliah et al., 1997; Champagne et al., 2002). Spatial learning and working memory, tested in the octagonal-arm radial maze, was impaired in ApoE^–/–^ mice at the age of 9–10 months (Evola et al., 2010). It is worth mentioning that ApoE^–/–^ and control mice were submitted to a rotarod test at 5–6 and 12–14 months of age, and both strains presented decreased average latency in the time they remained in the apparatus (because of aging), which means that ApoE deficiency was not associated with motor alterations (Fuentes et al., 2018).

The cholinergic system also seems to be affected in ApoE^–/–^ mice. The ChAT activity was reduced in the hippocampus and frontal cortex of 6-month-old ApoE^–/–^ mice (Gordon et al., 1995) and a significant decrease in AChE activity in the cortex, hippocampus, and septum in 14-week-old ApoE^–/–^ mice (Fisher et al., 1998).

Neuronal cell death markers (caspase-1 positive cells) increase in the hippocampus of ApoE^–/–^ mice when they are fed a high cholesterol diet (Rahman et al., 2005). The amyloid cascade is also disturbed in the brains of ApoE^–/–^ mice. The clearance of synthetic Aβ, which was injected directly into the brain parenchyma of ApoE^–/–^ mice, was impaired (Ji et al., 2001). On the other hand, the elderly ApoE^–/–^ mice did not have their brain Aβ deposition measured by Congo red staining, unlike in a traditional AD mouse model (Shnerb Ganor et al., 2018). The association between Aβ levels and ApoE deficiency needs to be studied further.

Another hallmark of neuroinflammation found in ApoE^–/–^ mice was an increase in GFAP in the hippocampus and corpus callosum (Crisby et al., 2004). Moreover, the disruption of BBB integrity was showed in very young ApoE^–/–^ mice (8 weeks of age) by the BBB extravasation of Evans blue dye (Methia et al., 2001). Hafezi-Moghadam et al. (2007) suggested that ApoE deficient mice have a progressive, age-dependent BBB leakage in the cortex and cerebellum.

Therefore, both genetic mouse models of hypercholesterolemia (LDLr^–/–^ mice and ApoE^–/–^ mice) present cognitive impairments early in life. These are associated with neuroinflammation, BBB disruption, and neurodegeneration, but not with increased brain Aβ deposition (Figure 2). As the models induced by hypercholesterolemic diet consumption, these genetic models present cerebral dysfunction as early as 3 months. Given that genetic forms of hypercholesterolemia (e.g., familial hypercholesterolemia) are highly prevalent in the general population, these animals are suitable for the study of neurodegenerative diseases, especially AD (Figure 2).

### Animal Models of Obesity and Its Comorbidities and Features of Neurodegenerative Diseases

Obesity is associated with chronic low-grade systemic inflammation (Gregor and Hotamisligil, 2011; Lumeng and Saltiel, 2011). The pathophysiological effects of obesity are observed not only in adipose tissue but also in other organs, including the brain (Alford et al., 2018). Evidence has suggested that obesity in midlife is a risk factor for AD in later life (Kivipelto et al., 2005; Beydoun et al., 2008; Whitmer et al., 2008; Chuang et al., 2016; Gottesman et al., 2017).

#### Neurodegenerative Diseases and Rodents Fed a High-Fat Diet

Increased consumption of high energy/high-fat food (i.e., over-nutrition), is considered a critical environmental causative factor of obesity (van Baak, 2013). Taking this into account, the most common experimental models to study the consequences of obesity are rodents fed high-fat diets (HFDs; Buettner et al., 2006, 2007). Nowadays, rodents exposed to HFDs are also widely used to evaluate the impact of obesity on the brain (Arnold et al., 2014; Underwood and Thompson, 2016; Nakandakari et al., 2019; Garcia-Serrano and Duarte, 2020).

Several studies have shown that the consumption of a HFD impairs critical brain areas that are involved in cognition, which are affected in AD (Arcego et al., 2016; Lizarbe et al., 2018; Nakandakari et al., 2019). There are several possible reasons why a HFD may lead to memory impairment. However, neuroinflammation seems to play a central role, because it is present in brain tissues involved with memory (Johnston et al., 2011; Verri et al., 2012; Kao et al., 2019). These changes in inflammatory markers are directly involved in the pathogenesis of AD. Thus, the study of rodents fed a HFD becomes an interesting approach for investigating many aspects of neurodegenerative diseases.

Experimental studies demonstrated impaired working memory, that is, decreased spontaneous alternation in the T-maze in 2-month-old mice fed an extremely HFD (60% fat for 17 days), as well as moderate HFD (45% fat for 8 weeks; Arnold et al., 2014). Young adult rats exposed to a HFD (58% fat) for 12–15 weeks presented spatial memory deficits in the spatial object recognition test (Underwood and Thompson, 2016). More recently, Denver et al. (2018) published a study where 7–14 weeks-old mice fed a HFD (45% fat) for different periods (from 18 days to 21 weeks) presented a sustained recognition memory impairment, evaluated at the novel object recognition task. Also, 24-month-old F344xBN F1 rats who were fed a HFD (60.3% fat) for just 4 days showed impaired long-term memory and partially impaired spatial memory (Spencer et al., 2017). McLean et al. (2018) pointed out that HFD caused a rapid decline in mice’s episodic memory. Specifically, memory deficits appeared to occur after 1 day’s exposure to a HFD (60% fat).

Memory impairments in rodents fed a HFD are associated with synaptic dysfunction and neuronal death. For instance, 3-month-old mice fed diets containing 45 or 60% fat for 6 months present synaptic degeneration, characterized by a reduction in the content of proteins located in synapses in the hippocampus and cortex (Lizarbe et al., 2018). Moreover, the exposure of young mice to a HFD (around 30% fat) for only 3 days caused an increase in hippocampal apoptotic molecular signals (i.e., decreased expression of Bcl-2 and increased expression of Bax; Nakandakari et al., 2019). AChE activity was reduced in brain areas such as the prefrontal cortex of rats fed a HFD (Morganstern et al., 2012).

Notably, Nakandakari et al. (2019) pointed out that a few days’ exposure to a HFD induced alterations in AD markers in the mice’s hippocampus. Mice fed a HFD for 3 days exhibited an increase in Aβ levels and an elevation of tau phosphorylation and total tau content in the hippocampus. Six-week-old C57BL/6NHsd mice fed a HFD supplemented with sugar in drinking water (42g/L, 12 weeks), presented increased expressions of both 4G8 and 6E10, indicating Aβ deposition, as well as an increase in the insulin-degrading enzyme (IDE), an endopeptidase responsible for Aβ degradation, resulting in decreased Aβ clearance. Furthermore, HFD supplemented with sugar in drinking water augmented phosphorylation of tau compared with the control diet (Kothari et al., 2017). Busquets et al. (2017) indicated that chronic exposure (weaning until 16 months of age) to a HFD led to the appearance of amyloid depositions in the brain of C57BL/6J mice, which therefore pointed to a potential model of sporadic AD.

High-fat diet consumption is also associated with motor abnormalities in rodents. After being given a HFD for 20 weeks, mice displayed reduced locomotion in the open field test and increased missteps in a vertical grid test. These changes were associated with TH depletion in the SN and striatum (Jang et al., 2017). Kao et al. (2019) recently demonstrated that mice fed a HFD (60% fat) for 20 weeks presented decreased locomotor function, loss of dopaminergic neurons in the SN, and dendritic spine density reduction. We observed that rats fed a HFD for 25 weeks presented a reduction in ventral tegmental area (VTA) TH levels and non-motor features such as depressive-like behavior (Bittencourt et al., 2020). Moreover, α- synuclein mRNA expression was significantly increased in C57BL/6J mice fed a HFD compared with the control group (Han et al., 2017).

Another critical point is that behavioral alterations in mice and rats fed a HFD were accompanied by neuroinflammation and BBB disruption. Levels of NF-κB, a major transcription factor that regulates inflammatory genes, were more expressed in the brains of mice fed a HFD (45% fat for 34 days) at 7–14 weeks of age (Denver et al., 2018). Additionally, IL-1β protein levels increased in the hippocampus of 4-week-old mice after being fed a HFD for 3 days (Nakandakari et al., 2019). Furthermore, the expression of TLR4 was elevated in the brains of HFD compared with the control group (Denver et al., 2018). The density of astrocytes and microglia was increased in the dentate gyrus and cortex of HFD-fed mice (45% fat for 18 days) (Denver et al., 2018). HFD exposure for 25 weeks in rats induced increased astrocytes and microglia density in the SN and VTA (Bittencourt et al., 2020).

The HFD also caused neuroinflammation in the nigrostriatal pathway, that is, astrogliosis and microgliosis in the SN and striatum (Kao et al., 2019). Finally, the increased neuroinflammatory process in HFD animals is related to enhanced permeability of BBB. An increased extravasation of Evans blue dye through the BBB and a disturbance of brain tight junction proteins were observed in mice fed a HFD for 8 weeks (Zhan et al., 2018). It was recently demonstrated that HFD feeding induced the higher entry of 14C-sucrose and 99mTc-albumin into the brains of mice, which indicates BBB disruption (Salameh et al., 2019). It is also important to note that a HFD also caused an increased neuroinflammatory response, increased brain concentration of Aβ species, and exacerbated behavioral deficits in APP/PS1 mice (Bracko et al., 2020).

**FIGURE 3:**
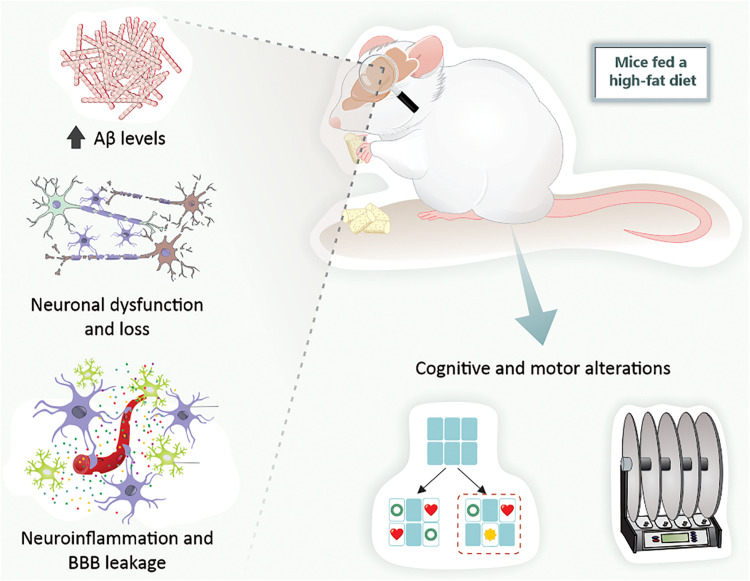
High-fat diet exposure causes cerebral alterations associated with neurodegenerative diseases in mice. Mice fed a high-fat diet present increased Aβ levels, neuronal dysfunction and loss, BBB leakage, neuroinflammation, and, ultimately, cognitive and motor alterations. Aβ, amyloid-β peptide; BBB, blood-brain barrier.

Taking all these findings into consideration, it can be verified that rodents exposed to a HFD exhibit the main hallmarks of both AD and PD. Indeed, just a short period of exposure to a HFD leads to brain alterations related to neurodegenerative diseases (Figure 3).

### Animal Models of Insulin Resistance and Type 2 Diabetes

Alzheimer’s disease is thought to involve insulin resistance and glucose hypo-metabolism (de la Monte, 2017; Mullins et al., 2017), but it is debatable whether these factors are triggers for neurodegeneration (Stanley et al., 2016). Human imaging studies demonstrate that glucose utilization by the brain declines with age and is notably impaired in subjects with early AD, which may be related to insulin action in key memory and cognition areas in the brain (Lee et al., 2018). Work on animal models of AD and T2D did show an association between dysfunctional insulin signaling in brain cells and AD-like pathology (Duarte, 2015; Lee et al., 2018). However, since glucose transport across the BBB into the brain parenchyma is not dependent on insulin (Duarte and Gruetter, 2012), it seems unlikely that typical glucose hypo-metabolism in AD is directly related to poor insulin sensitivity. On the other hand, insulin receptor activation stimulates signaling cascades for brain function regulation (Mullins et al., 2017). Insulin regulates the expression of genes necessary for memory consolidation (the MAPK/ERK pathway; Kelly et al., 2003; Dou et al., 2005), and also contributes to the control of the cellular metabolic sensor AMPK (Hardie, 2004; Marinangeli et al., 2016).

Diabetes mellitus takes two main forms: T1D results from inadequate insulin secretion, and T2D results from poor insulin action on target cells, that is, insulin resistance. Since T2D is highly heterogeneous, a refined classification of diabetes into five groups with different characteristics and risks of complications has been proposed (Ahlqvist et al., 2018). In any diabetic condition, insulin signaling perturbation impacts the brain, since insulin is involved in key processes such as metabolic regulation and synaptic plasticity (as reviewed in Duarte, 2015). Therefore, in addition to other metabolic syndrome factors such as hyperglycemia, dyslipidemia, hypertension, and vascular complications, impaired insulin signaling in diabetes likely contributes to the development of neurodegenerative disorders.

Several studies aiming to understand how diabetes impacts the brain have employed T1D models characterized by impaired insulin secretion and chronic hyperglycemia. The most extensively used T1D model is based on the administration of streptozotocin, which results in the destruction of β-cells and the halting of insulin production (Rees and Alcolado, 2005). Such treatment to either rats or mice results in a model with chronic severe hyperglycemia, causing neurotoxicity triggering memory deficits and impaired synaptic plasticity (Biessels et al., 1996), synaptic degeneration (Duarte et al., 2006, 2009), increased astrocyte reactivity and proliferation (Duarte et al., 2009), oxidative stress (Silva-Rodrigues et al., 2020), and altered brain metabolism (Duarte et al., 2009; Wang et al., 2012; Ruegsegger et al., 2019). Some of these findings have been reproduced in other T1D models that spontaneously develop diabetes due to the auto-immune destruction of β-cells, namely the non-obese diabetic (NOD) mouse (Saravia et al., 2002) and the BioBreeding/Worcester (BB/Wor) rat (Sima and Li, 2005).

Most available T2D models are associated with obesity (King, 2012). As is the case in diet-induced obesity models, spontaneous T2D rodent models with obesity display overt memory impairment and synaptic dysfunction as a result of a neurodegenerative process. This is so in polygenic strains such as the NONcNZO10/LTJ mouse (Duarte et al., 2012), the Otsuka Long-Evans Tokushima Fatty (OLETF) rat (Cho et al., 2020), and the Kuo Kondo mouse with agouti yellow (Ay) mutation (KK-Ay; Yin et al., 2017), as well as monogenic strains, namely the obese Zucker rat (fa/fa) that carries an autosomal recessive mutation of the fa-gene that encodes for the leptin receptor (Kamal et al., 2013), the leptin-deficient ob/ob mouse (Lepob/ob; Jeon et al., 2016), and the widely used db/db mouse that carries a spontaneous mutation in the leptin receptor gene (Leprdb/db; Chen et al., 2016; Zheng et al., 2016). Most relevant for understanding neurodegenerative pathologies is insulin resistance. When studying insulin resistance one can employ transgenic mice bearing gene deletions or mutations in genes required for insulin action and/or insulin secretion (Nandi et al., 2004). Such models, however, do not recreate a complete T2D phenotype.

There are very few non-obese T2D models available for research (King, 2012), and only the insulin-resistant Goto-Kakizaki (GK) rat has been probed for brain function (Duarte, 2015).

Goto-Kakizaki rats do not seem to show the overt deposition of Aβ in plaques or tau pathology that are typical of AD (Pereira et al., 2000; Candeias et al., 2017). However, their brains have an increased susceptibility to damage by stressors such as oxidative stress or aging (Duarte, 2015). Moreover, they display reduced neuronal glucose utilization and impaired glutamatergic neurotransmission, together with exacerbated mitochondrial astrocyte metabolism (Girault et al., 2019). In addition, glycogen metabolism in astrocytes, which is crucial for fueling glutamatergic neurotransmission and memory, was found to be impaired in insulin-resistant GK rats (Soares et al., 2019). Accordingly, it has been proposed that glycogen in cultured astrocytes is under insulin and IGF-1 regulation (Muhič et al., 2015).

These metabolic alterations in GK rats are accompanied by the development of synaptic dysfunction and increased astrocyte reactivity in the hippocampus, as well as spatial memory impairment (Duarte et al., 2019). In keeping with synaptic dysfunction in non-obese insulin resistance, activity between synapses was shown to trigger the mobilization of GLUT4 (the insulin-sensitive glucose carrier) from intracellular sources into axonal plasma membranes, a process that is mediated by the metabolic sensor AMPK. This is necessary to support the energy demands of active synapses (Ashrafi et al., 2017). Interestingly, it has been shown that toxic Aβ oligomers impair insulin signaling and decrease plasma membrane translocation of the insulin-sensitive GLUT4 in the hippocampus (Pearson-Leary and McNay, 2012). This might result in poor energy supply to neurons.

Despite astrogliosis (Duarte et al., 2019), neuroinflammatory microglia have not been yet reported in non-obese T2D models. Also, BBB leakage has not been confirmed in insulin resistance models, even though there have been reports of endothelial dysfunction (which impacts cerebral perfusion; see the discussion in Garcia-Serrano and Duarte, 2020).

## Conclusion

**FIGURE 4:**
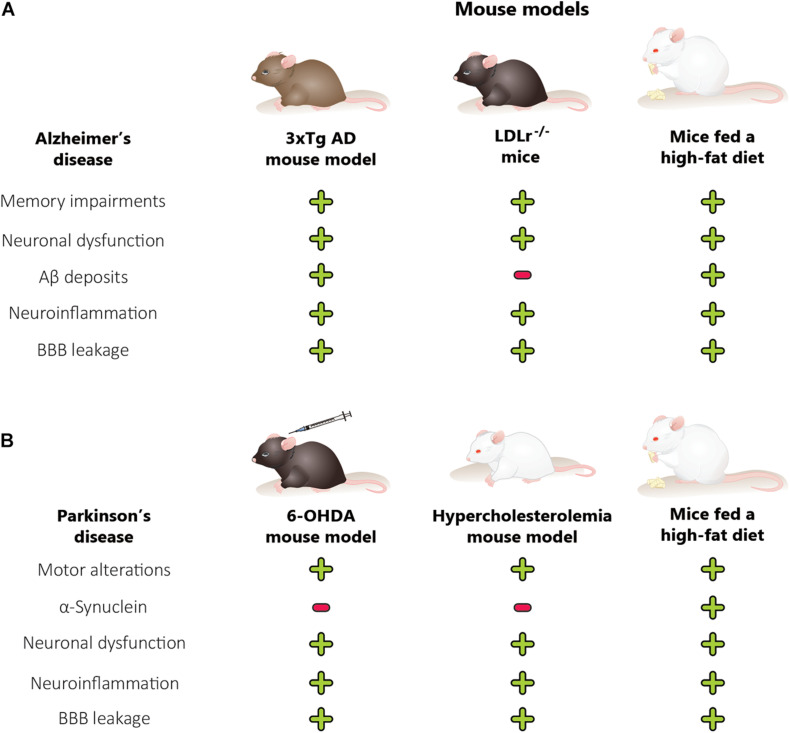
Comparison between animal models of metabolic disorders, Alzheimer’s disease mouse model and Parkinson’s disease mouse model. The features of Alzheimer’s disease **(A)**, including memory alterations, are also observed in hypercholesterolemic and obese rodents. The cognitive decline in obese and diet-induced hypercholesterolemic mice is associated with changes in Aβ levels, neuroinflammation, BBB dysfunction, and neuronal dysfunction, in the brain regions affected in Alzheimer’s disease. On the other hand, the genetic models of hypercholesterolemia also presented memory alterations, which are related to neurodegeneration, brain inflammation, BBB disruption, and neuronal dysfunction, but not modification in the Aβ deposits. Parkinson’s disease **(B)** features, including motor alterations, are also observed in hypercholesterolemic and obese rodents. Moreover, the hypercholesterolemic and 6-OHDA mouse model did not present an increase in α-synuclein mRNA expression while mice fed a high-fat diet presented. The motor alterations in obese, diet-induced hypercholesterolemic and 6-OHDA mice are associated with neuroinflammation, BBB dysfunction, and neuronal dysfunction in the brain regions affected by Parkinson’s disease. 6-OHDA, 6-hydroxydopamine; AD, Alzheimer disease; Aβ, amyloid-β peptide; BBB, blood-brain barrier; LDLr^–/–^, low-density lipoprotein receptor knockout mice. (+) There is alteration, (–) there is no alteration.

We can conclude that rodent models of obesity, diabetes, and hypercholesterolemia are useful tools for studying neurodegenerative disease development and characteristics. The main features of AD and PD, which include behavioral alterations such as memory and motor impairments, have been observed in hypercholesterolemic and obese rodents. The behavioral alterations in obese and diet-induced hypercholesterolemic mice are associated with Aβ content and α-synuclein changes, neuroinflammation, BBB dysfunction, and ultimately neuronal death in the brain regions affected by AD and PD (Figures 4A,B). In addition, genetic models of hypercholesterolemia have presented behavioral alterations (mainly those associated with AD) related to neurodegeneration, brain inflammation, and BBB disruption, but not modification in Aβ deposits (Figure 4A). Finally, brain alterations in mice and rats submitted to metabolic disorders occur earlier than in the classic rodent models of neurodegenerative diseases. The rodent models of metabolic disorders represent primarily the sporadic forms of neurodegenerative diseases.

## Author Contributions

All authors listed have made a substantial, direct and intellectual contribution to the work, and approved it for publication.

## Conflict of Interest

The authors declare that the research was conducted in the absence of any commercial or financial relationships that could be construed as a potential conflict of interest.
